# Numerical Investigation of the Damage Effect on the Evolution of Adiabatic Shear Banding and Its Transition to Fracture during High-Speed Blanking of 304 Stainless Steel Sheets

**DOI:** 10.3390/ma17071471

**Published:** 2024-03-23

**Authors:** Konstantina D. Karantza, Spyros A. Papaefthymiou, Nikolaos M. Vaxevanidis, Dimitrios E. Manolakos

**Affiliations:** 1 Laboratory of Manufacturing Technology, School of Mechanical Engineering, National Technical University of Athens, 15780 Athens, Greece; konstantinakarantza@mail.ntua.gr; 2 Laboratory of Physical Metallurgy, School of Mining and Metallurgical Engineering, National Technical University of Athens, 15780 Athens, Greece; 3 Department of Mechanical Engineering, School of Pedagogical and Technological Education (ASPETE), 15122 Amarousion, Greece

**Keywords:** adiabatic shear bands, shear localization, shear instability, damage criteria, thermal softening, damage softening, blanking, fracture, coupled analysis, LS-DYNA

## Abstract

This paper investigates numerically the effect of damage evolution on adiabatic shear banding (ASB) formation and its transition to fracture during high-speed blanking of 304 stainless steel sheets. A structural-thermal-damage-coupled finite element (FE) analysis is developed in LS-DYNA considering the modified Johnson–Cook thermo-viscoplastic model for both plasticity flow rule and damage law, while further, a temperature-dependent fracture criterion is implemented by introducing a critical temperature. The modeling approach is initially validated against experimental data regarding the fracture profile and ASB width. Next, FE simulations are conducted to examine the effect of strain rate and temperature dependence on damage law, while the effect of damage coupling is also evaluated, aiming to highlight the connection between thermal and damage softening and attribute them a specific role regarding ASB formation and transition to fracture. Also, the influence of dynamic recrystallization (DRX) softening is studied macroscopically, while further, a parametric analysis of the Taylor–Quinney coefficient is conducted to highlight the effect of plastic work-to-internal heat conversion efficiency on ASB formation. The results revealed that the implementation of damage coupling reacts to reduced ASB width and provides an S-shaped fracture profile, while it also decreases the peak force and results in an earlier fracture. Both findings are enhanced when accounting further for DRX softening and a higher value of the Taylor–Quinney coefficient. Finally, the simulations indicated that thermal softening precedes damage softening, showing that the temperature rise is responsible for ASB initiation, while instead, damage evolution drives ASB propagation and fracture.

## 1. Introduction

High-speed metal forming has concentrated significant interest in the construction industry, achieving high productivity and improved quality of final products. However, the enabled high strain rates can activate unstable plastic deformation mechanisms, which are strongly connected to dynamic failure. For this reason, a clear understanding of their evolution characteristics and their connection to fracture is necessary during tool design and optimization of process parameters. Specifically, adiabatic shear banding (ASB) reflects such an unstable deformation mechanism occurring under a high strain rate, leading to uncontrollable strain evolution and rapid fracture. In fact, ASB is initially manifested as deformed banding via severe shear strain localization along narrow bands, resulting in massive plastic work density, which is converted to internal adiabatic heat under the high strain rate. In consequence, the significant temperature increase inside ASB provides a transformed banding type, with dynamic recrystallization (DRX) and phase transformation being the two dominant mechanisms. Thus, ASB has been described as thermomechanical strain instability, which is reached when thermal softening overcomes strain hardening, reacting to catastrophic and rapid failure through micro-voiding and cracking propagation [1].

ASB plays an important role in the adiabatic blanking process of metal sheets, driving failure evolution and affecting the fracture profile. Chen et al. [2] investigated experimentally the microstructural evolution of ASB in fine-blanking of SS400 steel utilizing optical microscopy and scanning electron microscopy (SEM). Intense shear localization was developed initially around punch and die corners, leading to ASB formation. Highly elongated fine sub-grains were obtained within and alongside ASB, while the surrounding matrix consisted of an equiaxed cellular structure. Also, ASB revealed significantly high microhardness and massive temperature rise, showing a white-etching trace due to phase transformation. Finally, high hydrostatic pressure induced by a specially designed V-shaped blankholder and die reduced the extent of the fracture zone, improving the quality of the blanked surface. Hu et al. [3] experimentally studied C5191 phosphor bronze sheets under an ultra-high-speed blanking test utilizing electron backscatter diffraction (EBSD) and transmission electron microscopy (TEM) tools. The microstructural observations showed that the grains were stretched alongside the blanking direction, formulating strip-shaped structures, while the increase in graining misorientation led to fragmented grain layers. More, {001}<100> cube textures were formulated due to graining rotation, while the high enough adiabatic temperature rise also activated DRX in the blanked edge, consisting of distinct grain boundaries and a low dislocation density. Last but not least, sub-grains and high-angle boundaries were detected in the shear zone due to the twinning rotation. Sonkamble et al. [4] studied experimentally and numerically the influence of punch and die radii on shear localization in AA6082 by examining different radii for punch and die. The results indicated that shear strain was initially localized around punch–die corners, while a sharper punch of smaller radius showed greater maximum shear strain compared to a smoother die of slightly greater radius. Also, the shear strain was decreasing monotonically in the sheet interior, while the ASB core revealed highly elongated fine graining according to digital image correlation (DIC) experimental data for strain visualization. Finally, Fazily et al. [5] investigated AZ31B magnesium alloy via shearing tests for different clearance and temperature, utilizing optical microscopy and SEM, while micro-hardness Vickers tests were also conducted. In room temperature shearing, premature fracture was identified, revealing many defects such as micro-cracks, a jagged fracture profile, and loose particles, while a smoother blanked profile was provided as the temperature increased. Therefore, a higher temperature reduced the extent of the fracture zone, while lower clearance showed a decreased burnish zone extent. Eventually, shear banding together with twinning and DRX regions were obtained.

In addition, Winter et al. [6] investigated ASB evolution in adiabatic blanking of 22MnB5 steel by conducting parametric analyses regarding clearance, blanking velocity, and impact energy. Both experiments and finite element (FE) simulations in LS-DYNA were carried out, revealing S-shaped ASB for clearance below 6.67% of sheet thickness and velocity greater than 7 m/s. Also, lower clearance values reacted to increased compressive stress in the shear zone, which facilitated ASB formation together with DRX development, while instead, higher clearance required greater impact energy and velocity for ASB occurrence. Finally, an increased blanking velocity and low clearance provided longer and wider ASB, while the angle of the fractured surface was decreased. Gotoh et al. [7] studied the effect of shearing speed on shape quality for pure Al sheets, identifying that higher speed and low clearance provided the best shape quality. A 100 μm wide ASB was reported showing a recrystallized structure with even local melting, which facilitated fracture, resulting in a load drop. Schmitz et al. [8] analyzed the effect of strain rate sensitivity on ASB formation during adiabatic blanking. Quenched carbon C75S steel, bainitic HR760 steel, and quenched hardened 20MnB5 steel were examined by conducting both experiments and FE simulations. Strain rate sensitivity was indicated as the key factor for ASB width and microhardness, with 20 μm and 40 μm wide S-shaped ASBs obtained in 20MnB5 and C75S steels, respectively, while instead, HR760 steel did not show any ASB. Finally, lower strain rate sensitivity resulted in a more intense and narrower shear localization zone with earlier fracture initiation. Last but not least, Gaudillière et al. [9] studied C40 carbon steel at various blanking speeds from 5 to 20 m/s, revealing that greater speed results in lower blanking force through ASB formation, which reacts to less required energy for sheet cutting due to shear instability.

Further, Dodd [10] revealed that the peak blanking force depends on three modes of shear instability. In particular, the first two modes belong to geometrical instability due to process flow localization and microstructural evolution through voiding nucleation and growth, while the third mode refers to thermoplastic instability of material. The peak force was found to depend mainly on geometrical instability, showing that fracture precedes thermoplastic instability. Kolhatkar and Pandey [11] reported that cracking initiation reflects the blanking force collapse point, while a higher shearing speed strongly affects material plasticity due to a high strain rate and, in consequence, peak force. For this reason, their work highlighted the necessity for developing strain rate- and temperature-dependent FE models in order to study numerically the ASB mechanism during high-speed blanking. Additionally, Subramonian et al. [12] studied the influence of blanking speed on the peak force through FEM simulation in DEFORM 2D software, also underlining the importance of considering both the effects of temperature and strain rate on material modeling when accounting for shear localization aspects during the blanking process. Moreover, Bhoir et al. [13] summarized the utilized methods for optimizing process parameters, including experiments, FE simulations, neural network analysis, and genetic algorithms. In particular, Faura et al. [14] utilized the Cockroft–Latham ductile fracture criterion in FE simulations in order to assess optimum clearance, aiming for a clean, blanked surface. The cracking propagation path was predicted by linking the crack tips from the punch corner to the die corner. Moreover, Aravind et al. [15] indicated punch–die clearance as the most crucial parameter in fine blanking, suggesting that low clearance leads to a smoother blanked edge, while too-low clearance can cause tool misalignment and increased wear. Moreover, Sahli et al. [16] examined 16MnCr5 steel under fine blanking, reporting that low clearance slows fracture initiation, providing a more extensive sheared zone and a smoother blanked surface. Quazi et al. [17] described optimum clearance as the one that provides a diagonal cracking propagation along punch–die corners, reporting an optimization algorithm that considered Cockcroft–Latham damage criterion in BLANKSOFT software. Finally, Barik et al. [18] studied the effects of punch–die radii and clearance on the burr height of AA6085-T6 sheets by FE simulations in ABAQUS-CAE software. The Taguchi method was implemented in order to highlight the most critical parameter for burr formation, indicating that an increased punch radius and clearance lead to a higher burr height.

Also, several numerical studies have focused on the transition from ASB to fracture regarding the blanking process. Teng et al. [19] focused their work on differentiating ASB from fracture by conducting both experiments and FE simulations in ABAQUS. The arbitrary Lagrangian–Eulerian (ALE) adaptive meshing technique was implemented in order to restrict mesh distortion due to severe shear localization. The Bao–Wierzbicki fracture criterion was considered for assessing the damage extent, while the results indicated that periodical hot spots inside ASB act as micro-cracking initiators that propagate along the ASB and merge with each other. Marouani et al. [20] examined the blanking behavior of FeSi steel by carrying out parametric analyses of clearance and speed in ABAQUS. A Gurson–Tvergaard–Needleman damage model was applied for ductile failure accompanied by a strain rate-dependent material model. The results showed that increased speed results in lower peak force, as a high strain rate enhances adiabatic deformation and thermal softening. Myint et al. [21] studied the effect of blanking parameters on cut surfaces considering Cockcroft–Latham and Oyane damage models. The Oyane criterion proved more accurate for predicting the fractured profile, while clearance, punch–die radii, initial compression, punch diameter, and stress triaxiality were found to affect the quality of the cut surface. Moreover, Farzin et al. [22] examined numerically the influence of the die-to-punch radius ratio on crack initiation utilizing ABAQUS. Different damage criteria like the Gurson model, shearing failure model, and Goijaerts model were evaluated, revealing that the Gurson model seems to delay fracture while, in contrast, shearing failure predicts fracture initiation earlier than others. Yu and Zhao [23] utilized a modified Brozzo damage model accompanied by effective strain criteria in order to investigate strain and damage evolution during the fine blanking process, while Hambli and Potiron [24] considered the Chaboche–Lemaitre damage law during their study of blanking of 1060 steel sheets, proposing a critical damage parameter of 0.37 instead of 1, which is usually assessed. Finally, a modified nodal release method has been implemented in order to capture the cracking propagation path during FE simulation of high-speed blanking [25], while the GISSMO damage model has also been applied in high-speed impact shear-cutting simulations [26]. Summarizing, Table 1 contains the utilized experimental tools and damage criteria that have been studied during FE simulations in order to investigate the shear localization and the ASB formation during the blanking process.

Current work studies the effect of damage evolution on ASB formation and fracture during high-speed blanking of 304 stainless steel (304SS) sheets. A double structural-thermal-damage-coupled FE modeling is developed in LS-DYNA considering a thermo-viscoplastic material plasticity flow rule, while several forms of the modified Johnson–Cook damage criterion are examined, aiming to highlight the interaction between damage distribution and ASB evolution. In particular, strain rate and temperature dependence on damage law is evaluated, while further damage-coupled analysis is conducted by introducing the effect of damage extent on the stress and strain fields. Moreover, the macroscopic effect of DRX softening on ASB evolution and fracture is evaluated by utilizing an additional temperature-dependent fracture criterion, which introduces a critical temperature considering DRX. Also, a parametric study regarding the fraction of plastic work converted to internal heat is carried out, aiming to investigate its influence on ASB initiation through the evaluation of the thermal softening magnitude. Before the numerical simulation case studies, the developed modeling approach of fully structural-thermal-damage coupling is initially validated against experimental data regarding fracture profile and ASB width. The numerical results of simulation case studies are focused on assessing the effect of each damage criterion on the peak blanking force, ASB evolution, failure propagating rate, and fractured profile, while the distributions of strain, temperature, and damage fields are also discussed.

Finally, the parallel and conjugated implementation of both structural-thermal coupling and structural-damage coupling targets is conducted to identify the influence of both thermal and damage softening on ASB formation and its transition to fracture. Thus, the main focus of the current study is to investigate the competition between the two softening mechanisms during adiabatic blanking, revealing the one that is responsible for the genesis of ASB and the one that drives ASB evolution and its transition to fracture. In that way, this paper aims to attribute distinct roles to thermal and damage softening regarding ASB formation, which has remained a challenging task until now. In fact, the separation between the two softening mechanisms highlights more sufficiently the stages of ASB evolution and fracture, as well as their effect on the fracture profile and the required blanking force. Also, the investigation of the energy conversion efficiency from plastic work to internal heat remains crucial due to its effect on the thermal softening magnitude. Therefore, the parametric study of the fraction of plastic work converted to heat aims to reveal its influence on ASB and fracture, instead of assuming a constant value of 0.9 as suggested from earlier studies, which, however, attracts several concerns according to recent findings, which propose its variance with material, loading mode, and strain level [27].

## 2. Methodology

### 2.1. Examined Configuration

**Figure 1:**
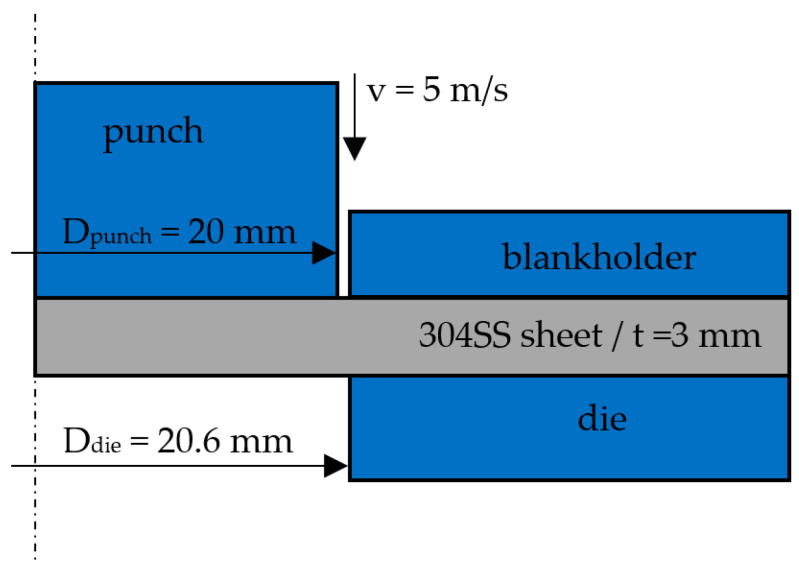
Examined blanking configuration.

This study investigates the effect of damage evolution on ASB formation and fracture of 304SS sheets during the high-speed adiabatic blanking process. FE numerical simulations are carried out in LS-DYNA software (Livermore Software Technology Corporation, Livermore, CA, USA) by utilizing LS-PrePost-4.3 for modeling development and post analysis. Figure 1 illustrates the examined blanking configuration, which consists of a cylindrical punch of 20 mm diameter, 304SS circular sheet 3 mm in thickness, and cylindrical blankholder and die of 20.6 mm internal diameters. In that way, a punch–die clearance of 0.3 mm is provided, which equals 10% of the sheet thickness and comes in sufficient agreement with relevant studies in the conventional blanking process [8]. Further, the half-axisymmetric domain is studied due to cylindrical geometries, while the punch, blankholder, and die are all considered rigid steel bodies through the *Mat020_rigid* material card in LS-DYNA. Finally, a constant blanking speed of 5 m/s was applied in FE numerical simulations in order to allow for developing adiabatic conditions during high-speed blanking, which is obtained at speeds greater than 3 m/s [6]. The constant velocity was attributed to the punch body via the *Prescribed_Motion_Rigid* card, while the termination time was set to 0.6 ms in the *Control_Termination* card in order to allow for full punch penetration. Therefore, the applied speed was selected sufficiently high to facilitate ASB formation by enforcing a high strain rate, which provides intense adiabatic heating and enhances the temperature increase inside the band, promoting shear instability due to thermal softening.

### 2.2. FE Modeling

#### 2.2.1. FE Discretization

**Figure 2:**
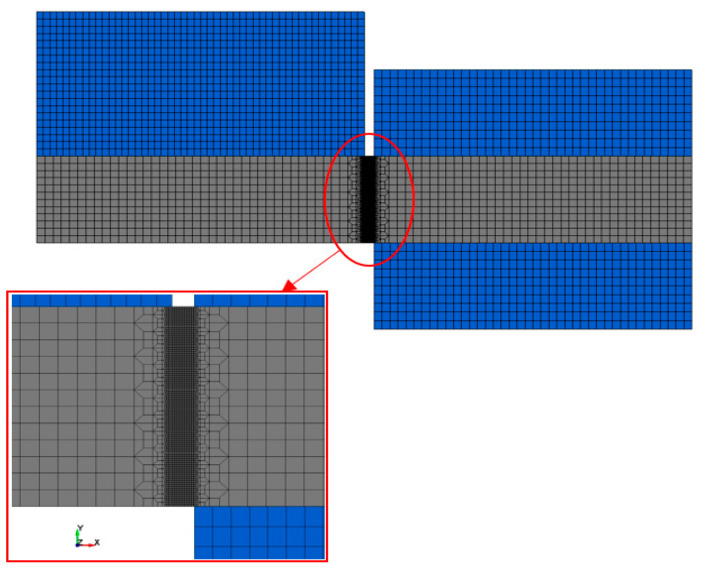
FE variable mesh with refinement in ASB expected region.

For investigating the effect of damage evolution on ASB formation and its transition to fracture during high-speed blanking of 304SS sheets, FE numerical simulations are carried out in LS-DYNA software. The half-axisymmetric domain is analyzed by implementing two-dimensional (2D) 4-node solid elements for reducing the computational cost, while a variable-density arbitrary Lagrangian–Eulerian (ALE) element meshing is utilized for the discretization of 304SS sheets. In fact, the 2D axisymmetric solid elements are adjusted via the *Section_Shell* card by setting the *ELFORM* parameter equal to 15, while the ALE element type is applied by the *SETYP* parameter equal to 3. Further, the FE mesh is intentionally refined to a 10 μm element size in the ASB expected region, which expands through sheet thickness and along the punch–die clearance, as shown in Figure 2. Instead, the surrounding sheet matrix is meshed with larger elements of 200 μm in size, as the strain field is diffused to zero, in contrast to the ASB core where intense shear strain localization appears. The shear-affected zone, which takes place between ASB and the surrounding matrix, is meshed via an intermediate microscale element size from 20 to 100 μm. In that way, a smooth transition from very fine meshing to a coarser one is achieved, preventing numerical instabilities. The very fine FE mesh within ASB’s expected region is necessary for capturing the occurrence of ASB and predicting its width, which has been estimated for about some decades in micrometers [1]. The ASB width is assessed through the visualization of shear strain distribution and obtaining the extent of critical strain transversely to the ASB direction.

Further, the ALE meshing type is implemented in order to avoid mesh distortion along ASB due to severe shear localization, which would decrease computational accuracy. Also, the Flanagan–Belytschko stiffness form for hourglass control is applied via a 0.1 hourglass coefficient by adjusting *IHQ* parameter equal to 4 in the *Hourglass* keyword of LS-DYNA, in order to prevent hourglass deformation and avoid numerical instabilities during computations due to volumetric blockage [28]. Although the Flanagan–Belytschko stiffness form supports shell elements, its implementation during FE modeling was feasible because of the 2D solid elements that are utilized.

Regarding the boundary conditions, a 2D surface-to-surface contact algorithm is considered for preventing the penetration between interacting interfaces in contact between the sheet and the rigid bodies. Moreover, a 2D single-surface contact algorithm is additionally adjusted to a 304SS sheet to avoid penetration between cracking edges in contact during the final stages of fracture. In each case, static and dynamic friction coefficients of 0.74 and 0.57, respectively, are applied considering steel-to-steel contacts according to the open literature data. Both contact types are implemented in the *Contact* keyword by modifying the *2D_Automatic_Surface_To_Surface* and *2D_Automatic_Single_Surface* cards, respectively, in order to determine the boundary conditions of no penetration in the interfaces. Specifically, the first contact algorithm treats the sheet as a deformable body, while the rigid bodies are considered undeformable. In contrast, the 2D single-surface algorithm considers only the sheet as a deformable body without setting any rigid surface, as its application refers to the contact between the interacting crack edges internally of the sheet material. Finally, static and dynamic friction coefficients are set via the *FS* and *FD* parameters, respectively.

#### 2.2.2. Material and Damage Modeling

For modeling the 304SS material’s mechanical behavior, a thermo-viscoplastic material flow rule is implemented through the modified Johnson–Cook (MJC) constitutive relation, which considers the effects of strain hardening, strain rate hardening, and thermal softening on material plasticity. The MJC material formula is implemented through the *Mat107-Modified_Johnson_Cook* material card in LS-DYNA, where all the material and damage properties are introduced. The MJC relation differs from the classic Johnson–Cook formula by the fact that it considers the strain rate hardening part as a power-law term instead of a logarithmic one. Further, a structural-thermal coupled analysis is developed during FE modeling in order to capture the thermomechanical macroscopic behavior of ASB. Specifically, except for the consideration of a thermo-viscoplastic material plasticity flow rule, the structural-thermal coupling takes into account the conversion of plastic work to internal adiabatic heat due to a high strain rate, which results in a significant temperature increase leading to thermal softening. For this reason, a Taylor–Quinney coefficient is applied, which represents the portion of plastic work converted to adiabatic heat. For evaluating the transition from ASB to fracture, the thermo-viscoplastic MJC damage model is implemented, which also considers the effects of strain, strain rate, and temperature on damage initiation and evolution. Except for the structural-thermal coupling, the developed FE models implement damage-coupled analysis by assessing the influence of damage extent on the stress and strain field, simulating in that way the effect of damage softening on material behavior.

In particular, the plastic flow stress *σ* is calculated according to the MJC flow rule formula, as described in Equation (1), composed of strain hardening, strain rate hardening, and thermal softening terms. *A*, *B*, *n*, *C*, and *m* refer to MJC material parameters, while r and $\dot{r}$ refer to the damage-equivalent plastic strain and strain rate, respectively. Also, $T^{*}$ refers to a homologous dimensionless temperature, which is computed through the material temperature *T*, room temperature *T_r_*, and melting temperature *T_m_*, as Equation (2) depicts. Moreover, ${\dot{r}}^{*}$ refers to the normalized damage-equivalent plastic strain rate, which correlates $\dot{r}$ with respect to the reference strain rate ${\dot{\varepsilon }}_{0}$, as shown in Equation (3).

(1) $$\sigma =(A+B\cdot r^{n})\cdot {\left(1+{\dot{r}}^{*}\right)}^{C}\cdot (1-{T^{*}}^{m})$$

(2) $$T^{*}=\frac{T-T_{r}}{T_{m}-T_{r}}$$

(3) $${\dot{r}}^{*}=\frac{\dot{r}}{{\dot{\varepsilon }}_{0}}$$

The damage coupling introduces r and $\dot{r}$, which are defined in Equations (4) and (5), respectively, and they are connected to the equivalent plastic strain ${\bar{\varepsilon }}^{p}$ and strain rate ${\dot{\bar{\varepsilon }}}^{p}$ by implementing damage parameter *D* and coupling parameter *β*. A full damage-coupled analysis is conducted when the *β*-parameter is set to 1, while a zero value allows for uncoupled analysis between damage and plasticity.

(4) $$r={\bar{\varepsilon }}^{p}(1-\beta \cdot D)$$

(5) $$\dot{r}={\dot{\bar{\varepsilon }}}^{p}(1-\beta \cdot D)$$

Also, the damage parameter *D* evaluates the damage magnitude leading to fracture via element erosion when it reaches a critical value *D_c_*, which is equal to 1. More specifically, the MJC damage criterion computes the fracture strain *ε_f_* as a function of stress triaxiality $\sigma^{*}$, normalized strain rate ${\dot{\varepsilon }}^{*}$, and homologous temperature ${Τ}^{*}$, as expressed in Equation (6). In that way, the MJC damage law represents a thermo-viscoplastic fracture criterion that is suitable for capturing the transition from thermomechanical ASB instability to dynamic failure. Moreover, *D*_1_, *D*_2_, *D*_3_, *D*_4_, and *D*_5_ refer to MJC damage parameters, while stress triaxiality $\sigma^{*}$ is defined as the ratio of hydrostatic pressure $\sigma_{H}$ to equivalent stress *σ_eq_*, as Equation (7) describes. The normalized strain rate ${\dot{\varepsilon }}^{*}$ refers to the correlated equivalent plastic strain rate to the reference strain rate, as shown in Equation (8), while Equation (9) expresses the coupling between damage and stress field, from which the damage-equivalent stress ${\tilde{\sigma }}_{eq}$ is assessed.

(6) $$\varepsilon_{f}=(D_{1}+D_{2}\cdot \exp(D_{3}\sigma^{*}))\cdot {\left(1+{\dot{\varepsilon }}^{*}\right)}^{D_{4}}\cdot (1+D_{5}{Τ}^{*})$$

(7) $$\sigma^{*}=\frac{\sigma_{H}}{\sigma_{eq}}$$

(8) $${\dot{\varepsilon }}^{*}=\frac{{\dot{\bar{\varepsilon }}}^{p}}{{\dot{\varepsilon }}_{0}}$$

(9) $${\tilde{\sigma }}_{eq}=\frac{\sigma_{eq}}{\left(1-\beta \cdot D\right)}$$

For assessing the damage parameter *D* and evaluating the fracture extent, the damage evolution Δ*D* is calculated via the fracture strain and equivalent plastic strain increment $\Delta {\bar{\varepsilon }}^{p}$, as Equation (10) describes, leading to fracture when reaching a critical value $D_{c}$, which equals 1. Also, an additional temperature-dependent fracture criterion is adjusted by introducing the DRX temperature *T_DRX_* as the critical temperature *T_cr_* leading to failure through element erosion [29]. That criterion is implemented in order to evaluate the effect of DRX softening on the transition from ASB to fracture. In fact, the effect of *T_DRX_* on damage evolution has been proposed to be strain rate-dependent [29], suggesting that *T_cr_* equals *T_m_* at a low strain rate, while at a higher strain rate, it becomes equal to *T_DRX_*. However, this study considers *T_cr_* equal to *T_DRX_*, as Equation (11) defines, because the examined strain rate is sufficiently high. In this study, *T_DRX_* is estimated at 43% of *T_m_*, which remains in agreement with relevant work [29]. Finally, when the DRX softening effect is not considered on damage evolution, *T_cr_* is set equal to *T_m_* in order to capture local melting, which leads to micro-voiding and micro-cracking genesis at the following [1].

(10) $$\Delta D=D_{c}\frac{\Delta {\bar{\varepsilon }}^{p}}{\varepsilon_{f}}$$

(11) $$T_{cr}=T_{DRX}$$

The temperature increase is provided by the conversion of plastic work to internal adiabatic heat due to the high strain rate. Therefore, the plastic work rate ${\dot{W}}_{p}$ computed from Equation (12) generates heat by a fraction that refers to the Taylor–Quinney coefficient *χ*, leading to a temperature increase. Thus, the temperature increase rate ∂*T/*∂*t* is described in Equation (13), in which *ρ* is the material density and *C_p_* is the specific heat.

(12) $${\dot{W}}_{p}={\dot{r}\tilde{\sigma }}_{eq}={\dot{\bar{\varepsilon }}}^{p}\sigma_{eq}$$

(13) $$\frac{\partial T}{\partial t}=\chi \frac{{\dot{W}}_{p}}{\rho C_{p}}$$

**Scheme 1:**
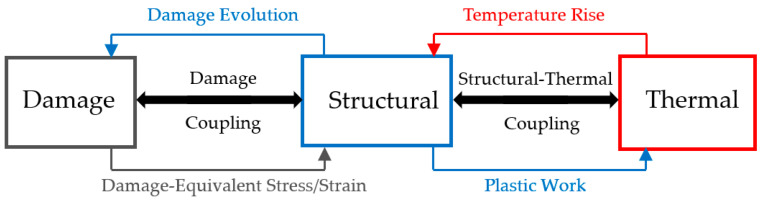
Structural-thermal-damage double coupling.

Table 2 contains the mechanical/thermal properties and the MJC material parameters of 304SS according to literature data [30,31]. Specifically, the strain rate parameters *C* and *D_4_* regarding the plastic flow rule and damage model, respectively, are correlated properly, as the ones provided from the literature refer to the classic Johnson–Cook formula, which is logarithmically strain rate-dependent. Also, Scheme 1 depicts a decomposition of the double coupled analysis conducted during the development of FE models, highlighting the interaction between stress/strain fields and temperature regarding structural-thermal coupling, as well as the interaction with damage regarding damage coupling. Specifically, the structural part of the model interacts with the thermal one by feeding it with the developed plastic work and receiving the estimated temperature increase from it, which is required to assess the thermal softening effect of flow stress. At the same time, the structural part computes the damage evolution, which provides damage-equivalent stress and strain.

**Scheme 2:**
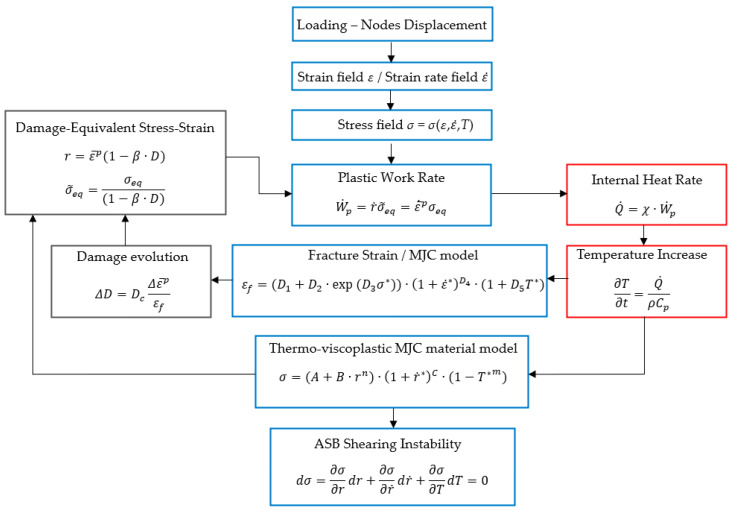
Flowchart of computational algorithm (blue boxes: structural part, red boxes: thermal part, grey boxes: damage part).

In more detail, the computational algorithm is briefly described in the flowchart depicted in Scheme 2. Therefore, the constant blanking velocity of 5 m/s provides a specific strain and strain rate field through nodal displacement, leading to a stress field whose plastic work generates internal heat by a percentage equal to the Taylor–Quinney coefficient. That heat is stored inside ASB due to a highly localized shear strain energy and high strain rate, which represents an adiabatic condition. Next, the temperature increase is calculated, neglecting heat diffusion due to the fact that the plastic work production rate is significantly larger than the heat diffusion rate due to the high strain rate, which also promotes adiabatic conditions [32]. The effect of temperature increment is considered on both the fracture strain and flow stress, while in the next step, the damage evolution is computed in order to evaluate the initiation and the extent of fracture. However, damage-equivalent stress and strain fields are introduced in the flow stress constitutive relation, affecting, in consequence, the provided plastic work. Finally, ASB shearing instability is obtained, as well as its transition to fracture, by evaluating the effect of both thermal and damage softening regarding the cause that triggers ASB initiation and the one that drives ASB evolution.

## 3. Modeling Validation

The developed modeling approach is initially verified against experimental data from previous work conducted by Schmitz et al. [8] in order to assess its validity and accuracy in predicting the ASB formation and the blanked profile. The utilized adiabatic blanking experiments examine quenched carbon spring steel C75S and quenched press hardening steel 20MnB5 subjected to high-speed blanking tests under 630 J impact energy, which provided an initial blanking speed of 5.8 m/s. Both steel sheets were 4 mm in thickness, while punch and die diameters of 29.84 and 30.1 mm, respectively, determine a relative clearance of 3.3%. For the needs of validating the procedure, two individual fully coupled models were developed according to the experimental set-up geometry and the tested materials, whose mechanical properties and Johnson–Cook parameters were selected based on the conducted work [8] and the open literature. Thus, the comparison between the developed models and the experimental data was made regarding the shape and straightness of the blanked profile and the ASB width. In particular, available measurements of blanked edge straightness and micrographs of the fracture surface, which reveal the ASB half-width, are taken into account for validating the numerical models. In fact, the validation against experimental data for 304SS was not achievable due to the lack of sufficient experimental data in case of high strain-rate blanking.

**Figure 3:**
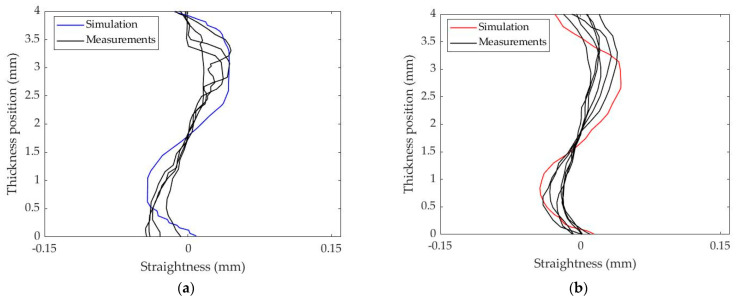
Blanked edge profile: (**a**) C75S steel; (**b**) 20MnB5 steel.

Figure 3 depicts the predicted blanked profile for C75S (blue line) and 20MnB5 (red line) steels compared to measurements (black lines) from adiabatic blanking experiments [8], showing that the numerical models sufficiently predict the S-shaped of the blanked profile as well as its deviation from straightness. More specifically, Table 3 quantifies the comparative results, revealing that the maximum range under which the blanked profile extends across the radial direction (*d_max_*) is predicted with errors of 7% and 10% for C75S and 20MnB5 steels, respectively. Also, the predicted ASB width agrees sufficiently with the one observed from the micrograph of the fracture edge, showing a deviation of 4 μm and 3 μm in the case of C75S and 20MnB5 steels, respectively.

**Figure 4:**
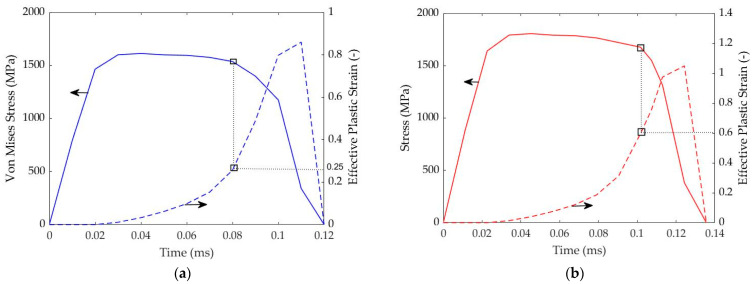
Stress (solid line) and effective plastic strain (dotted line) fluctuation inside ASB core: (**a**) C75S steel; (**b**) 20MnB5 steel.

**Figure 5:**
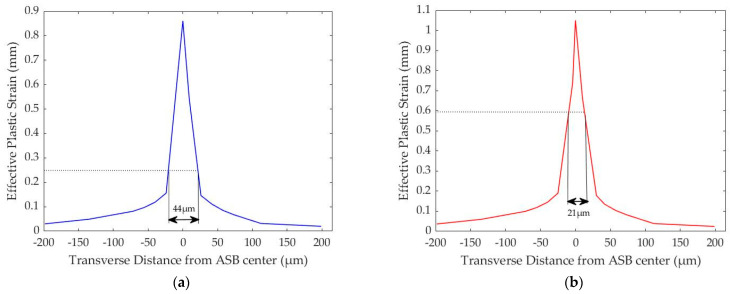
Transverse distribution of effective plastic strain: (**a**) C75S steel; (**b**) 20MnB5 steel.

In fact, the prediction of ASB width macroscopically has proven a challenging task due to the lack of microstructural data. For this reason, the macroscopic approach to assess ASB width is focused on obtaining the critical strain of stress instability that initiates ASB formation. Therefore, the ASB width can be determined from the lateral extent of the zone, which reveals a strain magnitude greater than the critical strain. In particular, Figure 4 shows the time fluctuation of stress and effective plastic strain inside the ASB core, revealing that stress instability provides a critical strain of 0.25 and 0.6 for C75S and 20MnB5 steels, respectively. Finally, Figure 5 illustrates the predicted transverse distribution of effective plastic strain for both steels, showing that C75S steel reveals a 44 μm wide ASB, which depicts a strain magnitude higher than the critical strain of 0.25, while 20MnB5 steel indicates a narrower ASB of 21 μm width, as captured from the extent of the zone with strain magnitude greater than the critical value of 0.6.

## 4. Results

### 4.1. Simulation Cases

The current study investigates the influence of damage extent on ASB formation and its transition to fracture during high-speed blanking of 304SS sheets. All study cases simulate the same blanking configuration depicted in Figure 1, while all models consider the thermo-viscoplastic MJC constitutive relation for material behavior. Regarding fracture criterion, all study cases make use of the MJC damage rule in various forms, while an additional temperature-dependent damage criterion is further implemented in parallel, considering a critical temperature equal to *T_DRX_* or *T_m_*. In fact, *T_DRX_* is considered the critical temperature when examining the effect of DRX softening on ASB evolution and fracture, while the other simulation cases consider *T_m_* for capturing local melting, which results in micro-voiding and micro-cracking genesis.

In more detail, Table 4 contains the simulation cases that are carried out aiming to evaluate the effects of temperature and strain rate, DRX, damage softening, and Taylor–Quinney parameters on the ASB evolution and fracture. In particular, regarding the examined form of the MJC damage criterion, three different scenarios are examined among the others. A single strain-dependent form is initially simulated considering only the effect of strain hardening (SH) on fracture strain, an augmented form that considers both SH and strain rate hardening (SRH) next, and finally, the full form of the MJC damage criterion (Full MJC), which considers the effect of SH, SRH, and thermal softening (TS) on fracture strain. Each term is represented in the fracture strain relation (Equation (6)) by the respective parenthesis; the SH term is determined from stress triaxiality and *D_1_*, *D_2_*, and *D_3_* damage parameters, the SRH term from strain rate and *D_4_* parameter, and finally, the TS term from the homologous temperature and *D_5_* parameter. Also, both damage-coupled (DC) and damage-uncoupled (DUc) simulations are conducted in order to evaluate the effect of damage softening on ASB propagation and fracture.

For the first study case, the effect of strain rate and temperature on damage evolution and ASB formation is studied, considering a typical value for the Taylor–Quinney coefficient equal to 0.9. In this comparative study case, damage coupling and DRX softening are not taken into account in order to highlight clearly the effect of strain rate and temperature. Therefore, the first study case compares the “Full MJC-DUc-*χ*09”, “SH-DUc-*χ*09”, and “SH/SRH-DUc-*χ*09” simulation cases. Subsequently, the effect of damage coupling is evaluated by considering a full MJC damage model and *χ*-value equal to 0.9, without accounting for the DRX effect. Thus, the second study case compares the “Full MJC-DC-*χ*09” and “Full MJC-DUc-*χ*09” simulation cases. Next, the effect of DRX softening on ASB evolution and fracture is investigated by comparing the “Full MJC-DC-*χ*09” and “Full MJC-DC-DRX-*χ*09” simulations for the third study case. Finally, a parametric study on the Taylor–Quinney coefficient is carried out for *χ*-values of 0.5-0.7-0.9 in order to assess its influence on thermal softening magnitude and how it affects its competition with damage softening during ASB evolution. The examined values of the Taylor–Quinney coefficient are selected according to previous reports regarding 304L stainless steel under shearing loading, which report that the *χ*-value can differ from the typical value of 0.9 [27]. Concluding, Table 5 summarizes the conducted simulation study cases.

### 4.2. Strain Rate and Temperature Effect

**Figure 6:**
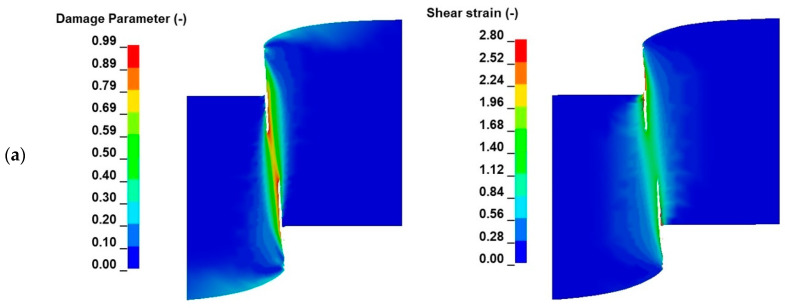

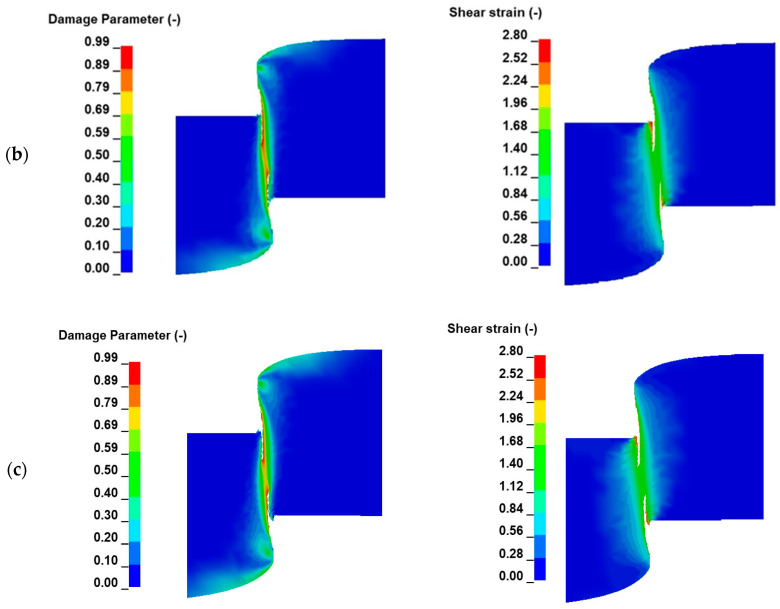
Damage and shear strain fields before final fracture: (**a**) Full MJC-DUc-*χ*09 at t = 222 μs; (**b**) SH-DUc-*χ*09 at t = 292 μs; (**c**) SH/SRH-DUc-*χ*09 at t = 302 μs.

This case study examines the effect of strain rate and temperature on ASB formation and its transition to fracture. Specifically, the “Full MJC-DUc-*χ*09”, “SH-DUc-*χ*09”, and “SH/SRH-DUc-*χ*09” models are compared in order to assess the influence of strain rate sensitivity and thermal softening on ASB initiation and evolution. Figure 6 depicts the damage and shear strain fields of the examined models at the final stage of ASB evolution and before the final fracture. The fields for each model refer to different timing (t), as each model treats damage evolution differently, and so it predicts final fracture at different times. In more detail, all simulation cases show a severe shear localization zone along the diagonal of punch-to-die corners. In particular, shear localization starts to develop around punch-to-die corners and expands through sheet thickness and along the maximum shear direction following. A comparison of the shear strain fields shows that the consideration of thermal softening on damage evolution predicts a narrower shear localization zone for the “Full MJC-DUc-*χ*09” model (Figure 6a) compared to the “SH-DUc-*χ*09” and “SH/SRH-DUc-*χ*09” models (Figure 6c and Figure 6b, respectively). That is because the temperature rise reacts to reduced flow stress, softening material plasticity and promoting shear instability, resulting in strain localization along the narrower high-temperature trace. Further, all cases reveal that cracking propagation follows the maximum damage direction, showing that damage evolution drives the cracking direction. Also, the implementation of temperature and strain rate dependence on the damage criterion shows that the damage is more localized around the crack tips, rather than uniformly distributed along ASB, as depicted in the damage fields of Figure 6a,c, respectively, thus controlling crack propagation.

**Figure 7:**
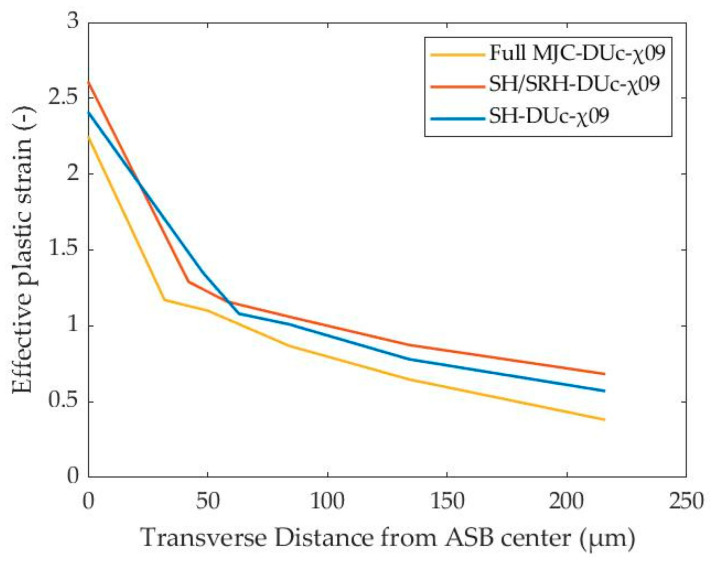
Effect of strain rate and temperature on the distribution of effective plastic strain transversely to ASB center before fracture.

Figure 7 also illustrates that the consideration of thermal softening leads to a narrower strain localization zone. However, the softened material plasticity due to temperature rise results in a lower peak strain due to earlier failure. In contrast, a higher maximum effective plastic strain is obtained when neglecting the influence of temperature, providing, however, a more diffused strain distribution, which is further enhanced when a single strain-dependent model is considered. Therefore, the implementation of strain rate and temperature dependence on damage evolution results in a narrower shear localization zone, thus reducing the ASB width.

**Figure 8:**
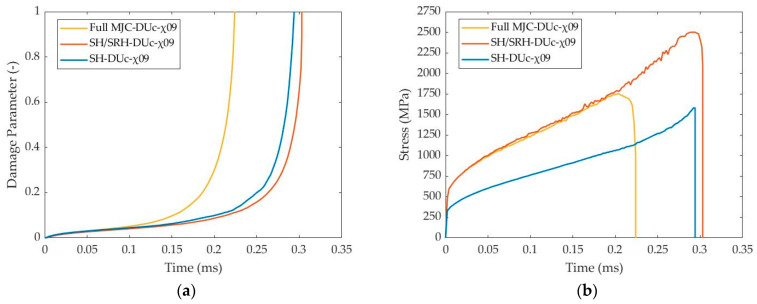
Effect of strain rate and temperature on damage evolution and flow stress inside ASB core: (**a**) damage evolution versus time; (**b**) flow stress versus time.

In addition, Figure 8a depicts the fluctuation of damage magnitude with time inside the ASB core, revealing that the implementation of thermal softening on the damage criterion predicts fracture initiation earlier. In particular, fracture inside the ASB core is manifested via a sharp increase in the damage parameter, which is predicted at about 210 μs from the “Full MJC-Duc-*χ*09” model, in contrast to the other models, which showed a damage increment in the range of 280 to 300 μs. Thus, thermal softening reacts to the earlier transition from ASB to fracture due to the fact that the provided temperature increase within ASB leads to micro-voiding through local melting, resulting in element erosion and earlier damage evolution. The time fluctuation of flow stress depicted in Figure 8b also confirms that the implementation of thermal softening on damage law reacts to earlier stress collapse due to micro-cracking initiation. In fact, stress collapse seems to coincide with a sharp damage increment, showing that damage evolution is the cause of rapid stress decrease, which reflects the fracture initiation through cracking failure. Also, homogenous plastic deformation was shown to be driven by strain rate hardening, which is confirmed by the concurrence between the “Full MJC-DUc-*χ*09” and “SH/SRH-DUc-*χ*09” models at early stages, while, when the magnitude of thermal softening starts to overcome that of strain rate hardening, strain instability is obtained, reflecting ASB generation.

**Figure 9:**
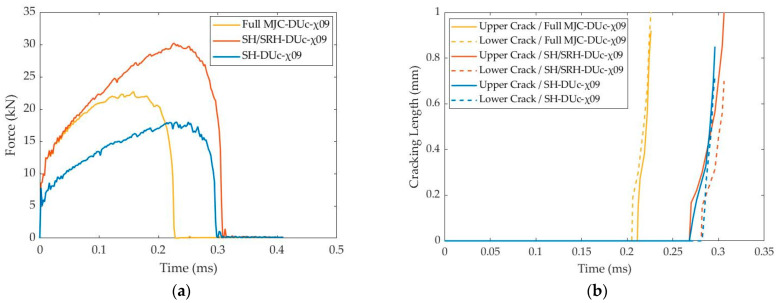
Effect of strain rate and temperature on blanking force fluctuation and cracking length: (**a**) force versus time; (**b**) cracking lengths (upper and lower).

Furthermore, the early deformation stages are driven by strain rate hardening as force fluctuation is confirmed in Figure 9a, while as deformation progresses and strain increases, the higher produced plastic work results in higher adiabatic heat and temperature rise, leading to a stronger thermal softening magnitude. Therefore, the peak force appears earlier when accounting for the thermal softening effect, also leading to a lower peak force due to softened material plasticity and reduced flow stress. In fact, the “Full MJC-DUc-*χ*09” model revealed a peak force of 22.7 kN, in contrast to the “SH-DUc-*χ*09” and “SH/SRH-DUc-*χ*09” models, which revealed 18 kN and 30.2 kN peak forces, respectively. Moreover, Figure 9b depicts the cracking lengths of the upper and lower cracks, which develop around the punch and die corners, respectively. The results reveal that the timing of sharp damage evolution coincides with cracking initiation and stress collapse, while the consideration of temperature dependence on the damage criterion shows an earlier fracture initiation via cracking genesis. Last but not least, the “Full MJC-DUc-*χ*09” model revealed that the lower crack developed around the die corner first, while the upper one generated around the punch corner later. On the contrary, the other two models showed that the upper crack initiated first, reaching a higher length, while the lower cracking length of the “Full MJC-DUc-*χ*09” model proved quite higher than those of the other models. Therefore, both upper and lower cracks seem to develop equally, reaching almost the same length when accounting for the thermal softening effect.

**Figure 10:**
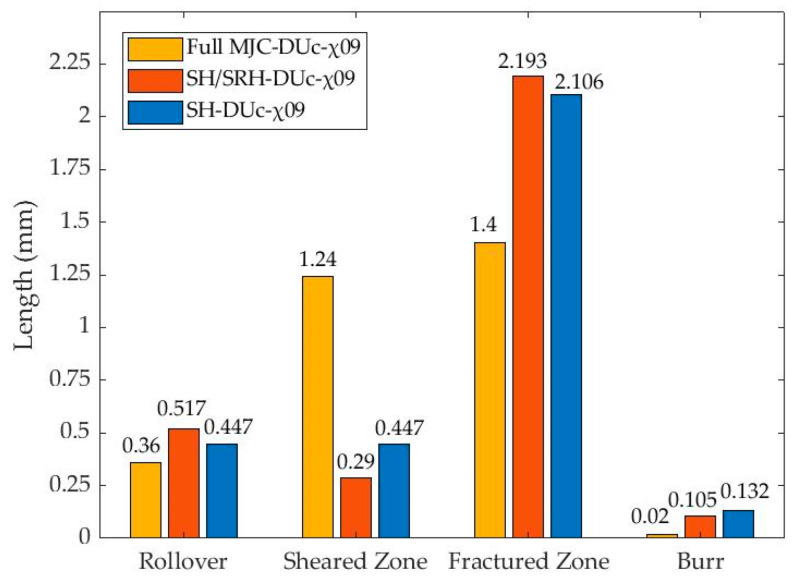
Effect of strain rate and temperature on the length of the district zones of blanked surface.

Finally, all three simulations of this case study revealed a conventional blanked surface profile in shape, in which the fractured surface extends more than the sheared one. In particular, Figure 10 depicts that the implementation of the thermal softening effect on damage evolution (“Full MJC-DUc-*χ*09” model) results in a slightly decreased rollover depth, while the lengths of the fractured and sheared zones are more equalized. On the other hand, when neglecting the temperature effect (“SH-DUc-*χ*09” and “SH/SRH-DUc-*χ*09” models), the fractured zone extends significantly more compared to the sheared edge, while burr formation is restricted below 100 μm.

### 4.3. Damage Coupling Effect

**Figure 11:**
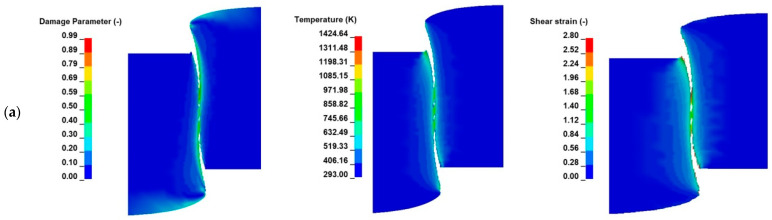

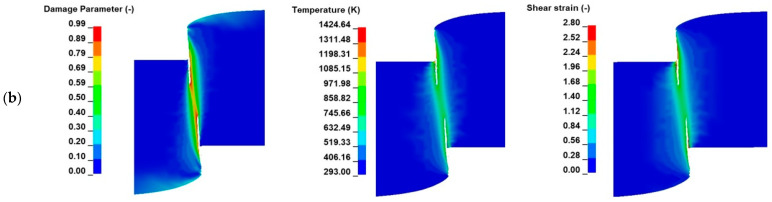
Damage, temperature, and shear strain fields before final fracture: (**a**) Full MJC-DC-*χ*09 at t = 166 μs; (**b**) Full MJC-DUc-*χ*09 at t = 222 μs.

**Figure 12:**
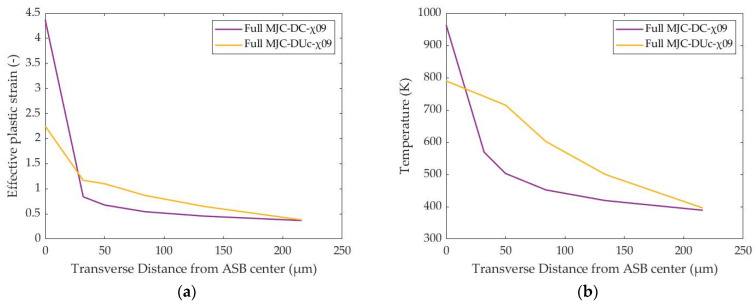
Effect of damage coupling on strain and temperature distributions transversely to ASB center before fracture: (**a**) effective plastic strain; (**b**) temperature.

This case study investigates the effect of damage coupling during numerical computations in order to evaluate the influence of damage softening on ASB evolution and fracture. For this reason, the “Full MJC-DC-*χ*09” and “Full MJC-DUc-*χ*09” models are compared in this part, with the first one accounting for damage softening through the coupling between damage evolution and stress/strain states. In more detail, Figure 11 depicts the damage, temperature, and shear strain fields during the final stage of ASB evolution and before the final fracture. Specifically, the implementation of damage coupling (“Full MJC-DC-*χ*09”) reveals earlier fracture at 166 μs instead of 222 μs (“Full MJC-DUc-*χ*09”), while further micro-voiding genesis is captured inside the ASB core as depicted in Figure 11a, leading to an intermediate micro-cracking formation except the upper and lower cracks. Also, damage softening results in a narrower high-temperature trace within the ASB core, which is accompanied by a more intense shear localization field, especially around crack tips. Moreover, the implementation of damage coupling seems to attribute damage evolution to a more S-shaped trace rather than a diagonal one, which is observed in the case of temperature evolution. Also, Figure 12a,b depict that the damage-coupled model reveals higher strain and temperature localization, as well as a greater peak strain and maximum temperature inside the ASB core. In contrast, more diffused strain and temperature distribution transversely to ASB are obtained when neglecting damage softening, showing that except thermal softening, damage evolution is also responsible for localizing the strain field and, in consequence, temperature rise, resulting in narrower ASB.

Therefore, damage coupling during numerical computations seems to provide a more intense and localized shear strain field, revealing narrower but stronger ASB while also accelerating the transition of ASB to fracture. Also, the implementation of damage coupling enhances the damage magnitude along the trace of ASB, in contrast to the damage-uncoupled numerical solution, which does not appear to have any internal fracture inside ASB even at a larger time. On the contrary, damage softening leads to internal fracture inside ASB, which is generated via micro-voiding through local melting due to higher temperature. Thus, the effect of damage softening seems strong on both ASB evolution and fracture, highlighting the reason for simulating damage-coupled models, as the influence of damage softening mechanism cannot be captured in the opposite case.

**Figure 13:**
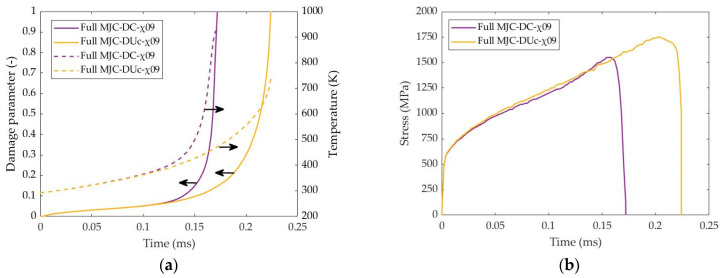
Effect of damage coupling on damage and temperature evolution and flow stress inside ASB core: (**a**) damage (solid line) and temperature (dotted line) evolution vs. time; (**b**) flow stress vs. time.

In addition, the damage and temperature evolution inside the ASB core is illustrated in Figure 13a, while Figure 13b shows the respective flow stress variance with time. The implementation of damage coupling during numerical computations clearly predicts an earlier fracture initiation, which is represented by a sharp increase in the damage parameter. Specifically, the “Full MJC-DC-*χ*09” model reveals a significant damage increment of around 170 μs instead of 230 μs, as provided by the “Full MJC-DUc-*χ*09” model. Also, Figure 13a shows that the significant temperature increase comes earlier than the timing of the damage sharp increment, indicating that thermal softening precedes damage softening. In that way, thermal softening can be described as the precursor of damage softening. That can be attributed to the fact that the massive temperature rise results in micro-voiding through local melting, leading to micro-cracking genesis and damage softening. Figure 13b shows that an earlier stress collapse is obtained when accounting for damage coupling, while a lower peak stress is also captured as both thermal and damage softening mechanisms are taken into account, facilitating shearing instability. More, Figure 13b ensures that stress collapse coincides with the timing of the sharp damage increment, indicating damage softening as the primary cause of stress collapse and thermal softening as the main reason for ASB initial formation. By that means, ASB genesis is attributed mainly to thermal softening, while instead, ASB evolution and its transition to fracture are driven by damage softening.

**Figure 14:**
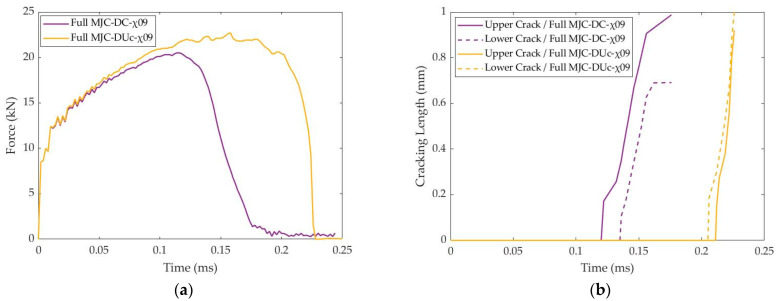
Effect of damage coupling on blanking force fluctuation and cracking length: (**a**) force versus time; (**b**) cracking lengths (upper and lower).

The peak blanking force also coincides with stress collapse timing at 170 μs, showing that the peak force is obtained when micro-cracking genesis takes place. Further, damage softening reacts to a peak force decrease of 8%, as the “Full MJC-DC-*χ*09” model reveals a 20.5 kN peak load, while the “Full MJC-DUc-*χ*09” model shows a maximum of 22.7 kN, as Figure 14a depicts. Figure 14b illustrates cracking propagation with time for the upper and lower crack developed around the punch and die corners, respectively. The results indicate that micro-cracks are generated earlier when accounting for damage coupling, while the upper crack appears to start developing earlier, reaching a greater length compared to the lower crack. On the contrary, the “Full MJC-DUc-*χ*09” model showed that both cracks develop almost at the same time. Finally, an S-shaped blanked profile is revealed from the “Full MJC-DC-*χ*09” model, rather than a conventional one with a district zone as provided by the “Full MJC-DUc-*χ*09” model, indicating that damage evolution determines the fracture profile, as damage evolution also revealed an S-shaped propagating trace for the “Full MJC-DC-*χ*09” model.

### 4.4. DRX Effect

**Figure 15:**
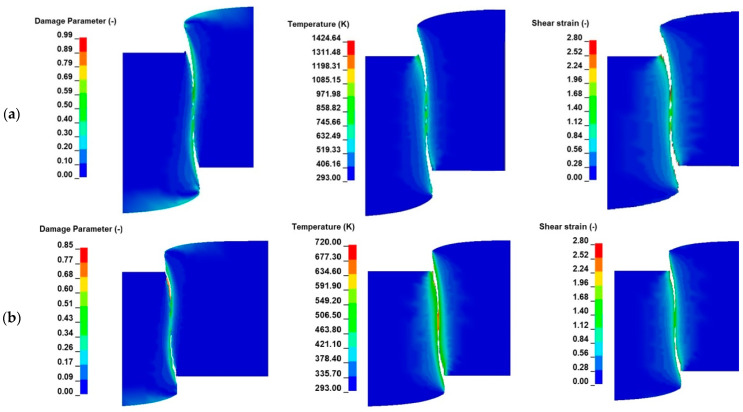
Damage, temperature, and shear strain fields before final fracture: (**a**) Full MJC-DC-*χ*09 at t = 166 μs; (**b**) Full MJC-DC-DRX-*χ*09 at t = 138 μs.

This case study investigates the effect of DRX softening from a macroscopic point of view by comparing the “Full MJC-DC-*χ*09” and “Full MJC-DC-DRX-*χ*09” models. Both models take into account damage coupling, but they differ in the critical temperature that is considered for fracture. Thus, the first model considers the melting temperature as the critical temperature, while the second model treats the DRX temperature as the critical one for fracture initiation, which is allowed due to the high-enough obtained strain rate of 10^5^ s^−1^ according to the simulation results. In more detail, Figure 15 depicts the damage, temperature, and shear strain fields during the final stage of ASB evolution and before fracture. The “Full MJC-DC-DRX-*χ*09” model predicts fracture earlier than the “Full MJC-DC-*χ*09” model due to element erosion when a DRX temperature of 720 K is reached. As shown in the temperature field of Figure 15b, the DRX temperature trace is located along the ASB core and seems to drive cracking propagation. On the other hand, a lower damage magnitude inside ASB is obtained when accounting for the DRX effect (“Full MJC-DC-DRX-*χ*09”), as the damage fields of Figure 15 confirm, indicating that DRX softening plays an important role in cracking propagation.

**Figure 16:**
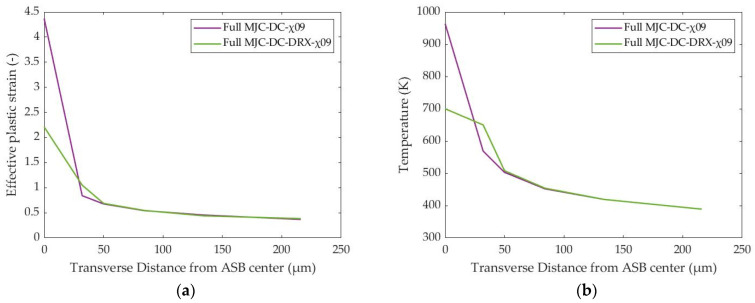
DRX effect on strain and temperature distributions transversely to ASB center before fracture: (**a**) effective plastic strain; (**b**) temperature.

Regarding the shear strain field, the implementation of the DRX effect seems to reveal a less intense strain field inside ASB, as element erosion due to DRX temperature does not allow for higher strain localization. For this reason, the peak strain within the ASB core reduces when accounting for DRX softening, as does the maximum temperature, because it is restricted to 720 K, while, furthermore, diffused stain and temperature distributions transversely to ASB are obtained, as Figure 16 shows. For this reason, significant attention must be paid when estimating the ASB width in case of considering the DRX effect. In the case of the “Full MJC-DC-DRX-*χ*09” model, a comparison between the transverse distribution of equivalent plastic strain and temperature shows that the heat-affected zone seems wider than the shear localization zone, as depicted in Figure 16a,b.

**Figure 17:**
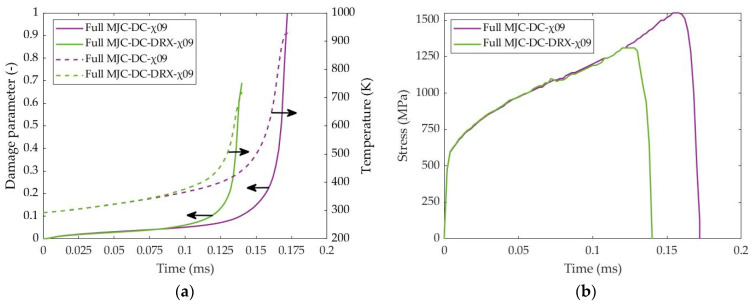
DRX effect on damage and temperature evolution and flow stress inside ASB core: (**a**) damage (solid line) and temperature (dotted line) evolution vs. time; (**b**) flow stress vs. time.

Figure 17a shows that the intense temperature increase precedes fracture initiation, which is manifested via the sharp damage increment. In fact, the “Full MJC-DC-DRX-*χ*09” model reveals both thermal and damage evolution earlier, indicating that DRX facilitates ASB development and reacts to premature fracture initiation. In particular, when DRX softening is accounted for, the fracture inside the ASB core occurs when the DRX temperature is reached, while damage evolution has not yet reached a critical value of 1, showing that DRX softening drives fracture. Last but not least, Figure 17b depicts that DRX softening further results in a lower peak flow stress, while stress collapse concurs with a sharp damage increment.

**Figure 18:**
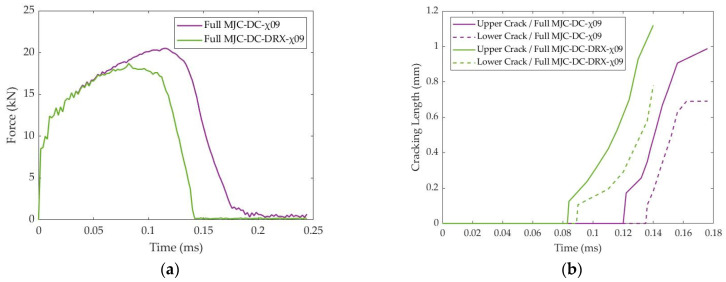
DRX effect on blanking force fluctuation and cracking length: (**a**) force vs. time; (**b**) cracking lengths (upper and lower).

Moreover, Figure 18a shows that DRX softening results in a lower peak force together with its earlier occurrence, which coincides with cracking genesis, as depicted in Figure 18b. In fact, the “Full MJC-DC-*χ*09” model provided a peak force of 20.5 kN at 116 μs, while the “Full MJC-DC-DRX-*χ*09” model showed a decrease in peak force of 9%, providing 18.6 kN at 84 μs. Both models reveal that an upper crack initiates first around the punch corner, while a lower crack formulates next. Also, both models predict that the upper crack reaches a higher length. However, an intermediate crack is formulated for the final fracture in both simulations. In fact, the implementation of the DRX effect on the critical temperature promotes element erosion at a lower temperature and before the magnitude of damage evolution becomes significantly strong, thus facilitating cracking genesis. For this reason, shearing instability is caused even earlier when accounting for the DRX effect apart from damage softening. Finally, both simulations reveal an S-shaped fracture profile in the blank sheet, showing that the DRX trace coincides with the damage softening direction.

### 4.5. Taylor–Quinney Parametric Study

The Taylor–Quinney coefficient describes the fraction of plastic work converted to internal heat, representing that way the connection between plastic deformation and temperature increases. In more detail, high plastic work under a large strain is converted to heat, which, under a high strain rate, does not have enough time to be diffused, and it is trapped inside ASB, leading to a significant temperature increase. In fact, a typical and constant *χ*-value of 0.9 has been globally considered from the majority of earlier studies; however, recent findings have reported that the *χ*-value can vary enough due to material, strain level, and loading mode [27]. In this final part, a parametric analysis of the Taylor–Quinney coefficient is carried out by examining *χ*-values of 0.5, 0.7, and 0.9. The examined range is based on experimental results regarding 304L stainless steel under dynamic loading [27]. For this reason, the “Full MJC-DC-*χ*09”, “Full MJC-DC-*χ*07”, and “Full MJC-DC-*χ*05” models are compared in this case study, aiming to evaluate the effect of energy conversion from plastic work to adiabatic heat. In that way, the influence of both thermal softening magnitude and thermal material behavior on ASB formation and fracture will be assessed. In more detail, the higher the Taylor–Quinney coefficient is, the higher the temperature increase becomes, as a greater amount of adiabatic heat is produced for the same plastic work. Thus, a higher *χ*-value promotes ASB formation by accelerating strain instability. However, the developed models do not consider the change in material mechanical and thermal parameters due to different temperature rise magnitudes, which are caused by the difference in *χ*-value.

**Figure 19:**
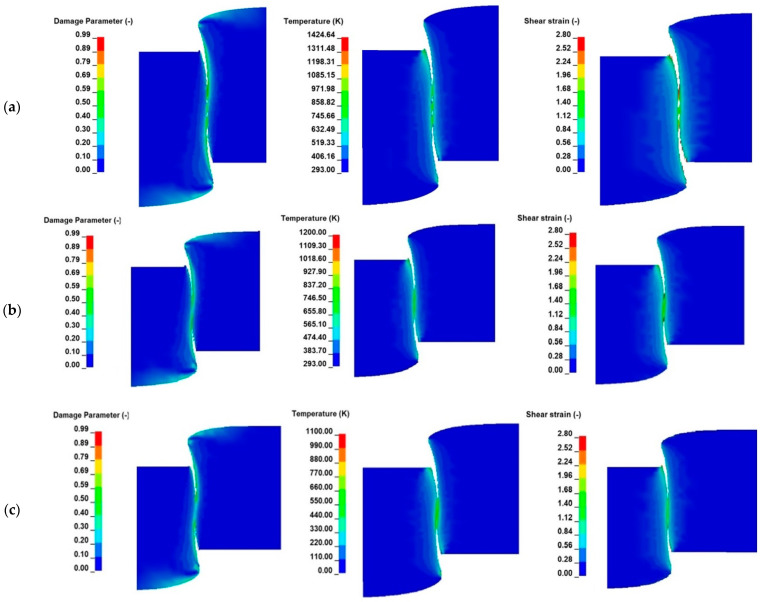
Damage, temperature, and shear strain fields before final fracture: (**a**) Full MJC-DC-*χ*09 at t = 166 μs; (**b**) Full MJC-DC-*χ*07 at t = 180 μs; (**c**) Full MJC-DC-*χ*05 at t = 180 μs.

**Figure 20:**
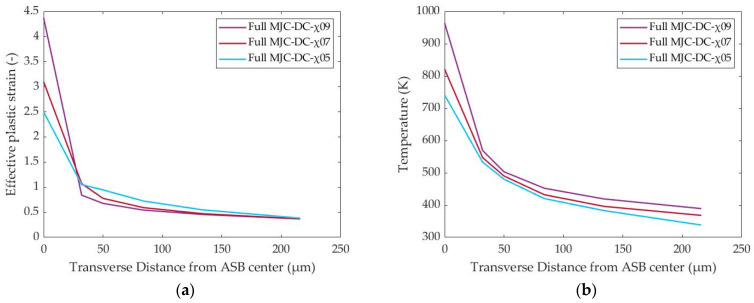
Effect of Taylor–Quinney coefficient on strain and temperature distributions transversely to ASB center before fracture: (**a**) effective plastic strain; (**b**) temperature.

As Figure 19 depicts, a higher *χ*-value results in a more intense temperature rise within ASB and, in consequence, a more advanced damage extent, while a *χ*-value of 0.9 shows even local melting inside the ASB core. That is because, for the same strain level, the produced internal heat is higher due to a greater *χ*-value, and so a higher ASB temperature is obtained under the same thermal properties of 304SS. Moreover, a higher *χ*-value reacts to more intense shear localization, also providing wider ASB. Figure 20 illustrates the transverse distribution of strain and temperature, confirming that a higher *χ*-value widens the ASB as a wider zone is revealed for the same critical strain. However, a greater temperature increase inside the ASB core leads to material degradation, facilitating strain instability and widening the ASB more. In fact, the temperature field of 0.5 *χ*-value seems almost to reach the DRX temperature, as depicted in Figure 20b, indicating that the activation of DRX softening depends strongly on plastic work-to-internal heat conversion efficiency. Therefore, *χ*-value above 0.5 can activate DRX softening inside ASB, reacting to maximum temperature marginally greater than the critical one of 720 K, and considering that 304SS shows *χ*-values higher than 0.5 [30], it is concluded that 304SS promotes DRX during ASB formation, which is not always guaranteed for every material [1].

**Figure 21:**
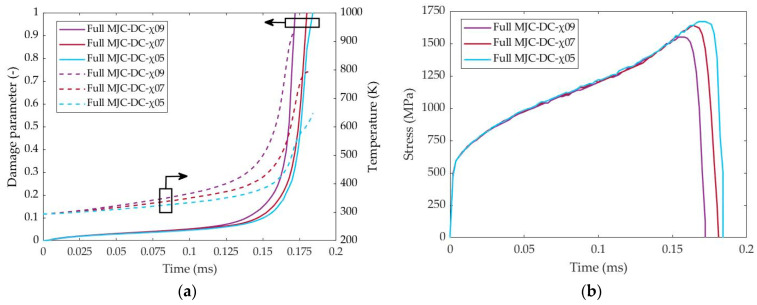
Effect of Taylor–Quinney coefficient on damage and temperature evolution and flow stress inside ASB core: (**a**) damage (solid line) and temperature (dotted line) evolution vs. time; (**b**) flow stress vs. time.

**Figure 22:**
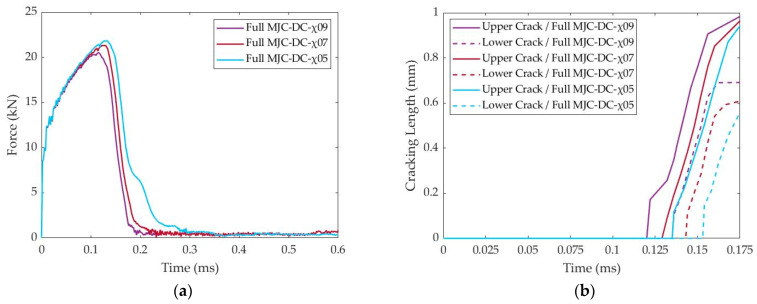
Effect of Taylor–Quinney coefficient on blanking force fluctuation and cracking length: (**a**) force versus time; (**b**) cracking lengths (upper and lower).

In addition, a higher *χ*-value results in an earlier temperature rise inside ASB, which allows it to reach a greater maximum temperature while it also activates the sharp damage increment earlier, thus facilitating fracture initiation, as confirmed in Figure 21a. In that way, a higher *χ*-value promotes both thermal and damage softening mechanisms, reacting to stress collapse sooner, as shown in Figure 21b. In fact, the stronger thermal softening magnitude, which is provided by the higher *χ*-value, results further in a reduced peak stress accelerating fracture initiation through critical damage, whose propagation is facilitated by the hotter and more softened ASB core. However, the higher *χ*-value does not seem to affect the stress progress before its peak value, as thermal softening has been found to affect mainly the ASB initiation by determining the starting point of strain instability instead of driving the fracture progress, which has been attributed to a damage softening mechanism. Finally, the increase in *χ*-value results in a lower blanking force, as well as an earlier fracture too, as shown in Figure 22a. In fact, *χ*-values of 0.5-0.7-0.9 provided peak forces of 21.8-21.3-20.5 kN, respectively, showing a mean decrease in peak force of 3% per 0.2 step in *χ*-value. The earlier predicted fracture for higher *χ*-value is attributed to the earlier cracking initiation due to stronger thermal softening, which can result in local melting and micro-voiding, leading to micro-cracking genesis. Figure 22b confirms that the “Full MJC-DC-*χ*09” simulation reveals sooner cracking evolution, while further, the upper crack forms first in all simulations, reaching a greater length. Finally, all three simulations revealed an S-shaped blanked profile, indicating that fracture is driven by damage softening, whose trace also follows a sigmoid trajectory.

## 5. Discussion

The structural-thermal-damage-coupled analysis developed in this work aims to investigate the formation of ASB and its connection to fracture during the high-strain-rate blanking process of 304SS. The thermo-viscoplastic MJC formula is implemented for both material flow rule and damage law, while further damage equivalent stress and strain fields are introduced. The double coupling between structural-thermal and structural-damage fields aims to evaluate the contribution of thermal and damage softening mechanisms on ASB genesis and evolution. In the current work, the analysis is organized into four main case studies by investigating the influence of strain rate hardening and thermal softening on both the plasticity flow rule and damage law, the effect of damage coupling, the impact of DRX softening, and finally, the Taylor–Quinney coefficient.

More specifically, accounting for the thermal softening effect during numerical computations showed a lower peak force under earlier fracture, while the effect of temperature rise seems to equalize the extent of shear and fracture zones. On the contrary, a significantly larger fracture zone is obtained when neglecting the contribution of temperature increase. In that way, the consideration of temperature rise due to the increased plastic work under adiabatic conditions, which are secured by a high strain rate, is of significant importance when assessing the required blanking force or aiming to minimize the fracture zone extent for improving product quality.

In addition, the importance of considering damage coupling seems crucial, as it provides an S-shaped fracture profile during high-speed blanking rather than a conventional blanked profile with district zones, which is observed more in quasi-static conditions. Also, damage coupling revealed an 8% drop in peak force and earlier fracture, indicating that its neglect during simulations can lead to overestimating the required load capacity of the pressing machine or the required punch stroke. Moreover, simulations showed that thermal softening precedes damage softening, indicating that the first one is responsible for initiating ASB, while damage softening drives ASB evolution and its transition to fracture. In fact, both thermal and damage softening are manifested via the sharp increase in temperature and damage evolution, respectively. The numerical results revealed that the peak blanking force coincides with the sudden stress collapse, which is attributed to the significant damage increment. By that means, the evaluation of both thermal and damage softening mechanisms is necessary in order to predict accurately the fracture progress. In particular, the parallel and conjugated computation of strain failure, damage evolution, and critical temperature allows for estimating sufficiently the transition from ASB to fracture and, in consequence, the predicted blanked profile.

Further, the macroscopic assessment of the DRX effect on ASB and fracture seems crucial, as it reacts to earlier material failure before the critical damage is reached. That is because a critical DRX temperature leads to premature fracture at high strain rates, revealing an extra 9% decrease in peak force when accounting for DRX softening. On the other hand, the blanked profile retains its S-shape as the DRX trace seems to follow the sigmoid trajectory of damage evolution. In fact, an augmented DRX damage criterion, which would consider the critical temperature as a function of DRX temperature, melting point, and strain rate, would enhance even more the accuracy of the model on capturing the influence of DRX, which remains more a microstructural phenomenon in its core.

Also, an accurate assessment of the *χ*-value seems very important, as it affects the temperature rise and, in consequence, the magnitude of thermal softening. In that way, the *χ*-value plays an important role in the determination of ASB genesis and the initiation of strain instability. In more detail, a higher *χ*-value results in earlier and more severe ASB, leading to a greater peak strain and temperature, while it further widens the shear band. The increased *χ*-value also reduces the peak force by a rate of 3% per 0.2increase in the *χ*-value. Therefore, the actual evaluation of the *χ*-value has proved crucial in predicting the peak force and ASB evolution, as its typical value of 0.9, which was globally considered during early studies, must be reassessed based on recent findings that highlight its influence from the material and the loading mode [27]. Finally, considering that the DRX temperature of 304SS is estimated at about 720 K and that the minimum examined *χ*-value of 0.5 for 304SS provides a maximum temperature marginally greater than 720 K, it is indicated that 304SS promotes DRX activation during ASB formation, which is not always guaranteed [1], underlining the importance of accounting for DRX softening effect during numerical computations.

Therefore, the implementation of damage coupling during numerical computations plays an important role in the evaluation of peak blanking force and blanked profile, which are strongly connected to the final product quality. Also, the DRX effect and *χ*-value are both key factors in predicting accurately the ASB evolution and its transition to fracture, as they affect the temperature field and the failure progress. Capturing the temperature and damage evolution closely during simulations of high-speed blanking allows for accurate computations of the peak force and the effective punch stroke, as well as cracking initiation and propagation. In that way, the developed modeling approach can be applied to simulate either high-speed forming or machining processes in general, aiming to analyze the ASB mechanism and the way it affects the load capacity, product quality, material defects, and forming limits through its interaction with fracture. Moreover, simulating ASB propagation can also contribute to the field of tool optimization in order to prevent ASB and dynamic failure phenomena, decreasing material defects. However, the implementation of the developed coupled analysis is limited only to high-strain-rate applications, which remain a key prerequisite for ASB formation. In fact, the developed analysis presupposes adiabatic loading conditions under a high strain rate, thus neglecting heat diffusion and assuming that the provided internal heat from the plastic work is consumed by increasing material temperature. Therefore, applications under quasi-static conditions, which would allow enough time for heat diffusion, would require more accurate heat conduction computations. Also, the determination of MJC material and damage parameters often remain often a challenging task, especially when considering their temperature dependence, which, however, may be taken into account by implementing the tabulated Johnson–Cook material model. Finally, the continuous re-computation of the *χ*-value during simulation progress would increase even more the validity and accuracy of the numerical solution due to the dependence of the *χ*-value on strain magnitude.

## 6. Conclusions

This work developed a structural-thermal-damage-coupled FE analysis in order to investigate the effect of damage evolution on ASB formation and its transition to fracture during adiabatic blanking of 304SS sheets. The double coupling between structural-thermal and structural-damage state fields aims to highlight the influence of both thermal and damage softening mechanisms on initiating ASB and driving its propagation, leading to fracture. For this reason, the thermo-viscoplastic MJC flow rule was implemented for material plasticity, while different forms of the MJC damage law were examined together with a temperature-dependent damage criterion, which considers a critical temperature for fracture initiation. Subsequently, the main conclusions that are derived from the simulation results can be summarized as:

1. The consideration of thermal softening showed an earlier fracture initiation via cracking propagation, resulting in an earlier and lower peak force. Also, cracking initiation seems to be mainly determined by thermal softening, which revealed that upper and lower cracks appeared almost simultaneously. On the other hand, when neglecting the temperature effect, the upper crack manifested earlier, while only strain rate does not seem to affect the timing of cracking initiation. On the other hand, the strain rate was found to drive the early deformation stages, during which the temperature increase was weak, thus determining plasticity progress. Finally, the implementation of thermal softening showed a similar extent of sheared and fractured zones in a blanked profile regarding the damage-uncoupled models, while in contrast, the fractured zone was found to be significantly longer compared to other zones when considering only the strain rate hardening.
2. The consideration of damage coupling resulted in a reduced ASB width and higher maximum strain and temperature inside the ASB core due to additional material softening from the damage extent. For this reason, an 8% lower peak force was also revealed when accounting for both thermal and damage softening, while its occurrence was also captured sooner. In particular, damage coupling showed earlier fracture initiation, which was manifested via earlier cracking initiation around the punch and die corners. Finally, the fracture was completed through the formation of an intermediate micro-crack generated by micro-voiding within the ASB core.
3. Thermal softening was found to precede damage softening, indicating that temperature increase is responsible for ASB initiation, while damage evolution drives ASB propagation and its transition to fracture. For this reason, both peak force and stress collapse coincide with the timing of the sharp damage increment. In other words, strain instability seems to be generated from the significant temperature increase, while instead, its unstable evolution is primarily driven by damage softening. Finally, damage coupling revealed an S-shaped fracture profile due to the sigmoid trace of damage evolution, in contrast to the diagonal trace of temperature rise. Instead, the damage-uncoupled models revealed a conventional blanked profile with district zones.
4. DRX softening revealed an earlier cracking genesis and premature fracture, resulting in a further 9% peak force reduction. In fact, the fracture was obtained when reaching the DRX temperature and before critical damage in specific points of the ASB core, indicating that failure is driven by both critical temperature and critical damage. However, the maximum temperature inside ASB was found to be lower due to premature element erosion. Therefore, the implementation of multiple damage criteria in parallel seems very important, offering the capability to evaluate the influence of strain failure, damage evolution, and critical temperature on fracture. Finally, DRX softening also led to an S-shaped blanked profile, showing that DRX evolution follows the trace of damage increment.
5. A higher *χ*-value resulted in a wider thermal affected zone, providing greater temperature inside ASB. In fact, a *χ*-value of 0.9 resulted in micro-voiding generation due to local melting within the ASB core, in contrast to lower examined *χ*-values, which did not provide a high enough temperature to reach the melting point. Also, a higher *χ*-value reacted to a stronger thermal softening magnitude, which led to earlier ASB formation and damage increment, revealing a lower peak force. Specifically, a reduction rate of 3% in peak force was revealed per 0.2 increase step in *χ*-value. Moreover, the increase in *χ*-value results in wider ASB consisting of more intense shear localization, as confirmed by the greater peak strain. Finally, longer upper and lower cracks were observed for a higher *χ*-value, showing that a greater work-to-heat conversion efficiency promotes cracking propagation.
6. The lower examined *χ*-value of 0.5 revealed a maximum ASB temperature that was marginally greater than the DRX temperature of 720 K. Considering that 0.5 defines the lower bound of *χ*-value regarding 304SS and that the increase in *χ*-value results in a higher temperature rise, it is indicated that 304SS promotes the activation of DRX during ASB formation, which is not always guaranteed. Therefore, the implementation of DRX softening during numerical computations seems even more important in order to provide an accurate prediction of the strain instability point and fracture progress.

## Figures and Tables

**Table 1:** Experimental methods and damage criteria in FE simulations during analysis of ASB formation and fracture in high-speed blanking.

Experimental Method	References	Damage Criterion	References
Optical Microscopy	[2,5,6]	Cockcroft–Latham	[8,14,17,21]
Scanning Electron Microscopy	[2,5]	Bao–Wierzbicki	[19]
Transmission Electron Microscopy	[3]	Gurson–Tvergaard–Needleman	[20]
Electron Backscatter Diffraction	[3]	Oyane	[21]
Digital Image Correlation	[4]	Gurson	[22]
		Goijaerts	[22]
		Brozzo	[23]
		Chaboche–Lemaitre	[24]
		Modified Nodal Release	[25]
		GISSMO	[26]

**Table 2:** Mechanical-thermal properties and MJC material parameters of 304SS.

Mechanical and Thermal Properties		Constitutive Relation		Damage Criterion	
*ρ* (kg/m^3^)	7900	*A* (MPa)	310	*D_1_*	0.53
*E* (GPa)	200	*B* (MPa)	1000	*D_2_*	0.5
*ν* (-)	0.3	*n* (-)	0.65	*D_3_*	−6.8
*Cp* (J/kgK)	440	*C* (-)	0.0515	*D_4_*	−0.015
*a* (μm/m °C)	17.2	*m* (-)	1	*D_5_*	0.002
${\dot{\varepsilon }}_{0}$ (s^−1^)	1	${\dot{\varepsilon }}_{0}$ (s^−1^)	1	*D_c_*	1
*χ* (-)	0.9	*T_m_* (K)	1673	*T_DRX_* (K)	720
		*T_r_* (K)	293		

**Table 3:** Comparative results of validating C75S and 20MnB5 steel experiments.

	C75S		20MnB5	
	Experiment	Simulation	Experiment	Simulation
*d_max_* (μm)	100	93	85	94
Blanked profile shape	S-shape	S-shape	S-shape	S-shape
ASB width (μm)	40	44	18	21

**Table 4:** Simulation cases.

Simulation Case	Description	MJC Damage Criterion	Damage Coupling	DRX Softening	Taylor–Quinney, *χ* (-)
I	Full MJC-DC-*χ*09	Full ^1^	Yes	No	0.9
II	Full MJC-DUc-*χ*09	Full	No	No	0.9
III	SH-DUc-*χ*09	SH	No	No	0.9
IV	SH/SRH-DUc-*χ*09	SH + SRH	No	No	0.9
V	Full MJC-DC-DRX-*χ*09	Full	Yes	Yes	0.9
VI	Full MJC-DC-*χ*07	Full	Yes	No	0.7
VII	Full MJC-DC-*χ*05	Full	Yes	No	0.5

^1^ Full = SH + SRH + TS.

**Table 5:** Simulation study cases.

Study Case	Content	Simulations in Comparison
1	Strain rate/temperature effects	II-III-IV
2	Damage coupling effect	I-II
3	DRX effect	I-V
4	Taylor–Quinney coefficient effect	I-VI-VII

## Data Availability

Data are contained within the article.
